# Shorter treatment for minimal tuberculosis (TB) in children (SHINE): a study protocol for a randomised controlled trial

**DOI:** 10.1186/s13063-018-2608-5

**Published:** 2018-04-19

**Authors:** Chishala Chabala, Anna Turkova, Margaret J. Thomason, Eric Wobudeya, Syed Hissar, Vidya Mave, Marieke van der Zalm, Megan Palmer, Monica Kapasa, Perumal K. Bhavani, Sarath Balaji, Priyanka A. Raichur, Anne-Marie Demers, Graeme Hoddinott, Ellen Owen-Powell, Aarti Kinikar, Philippa Musoke, Veronica Mulenga, Rob Aarnoutse, Helen McIlleron, Anneke Hesseling, Angela M. Crook, Mark Cotton, Diana M. Gibb, on behalf of the SHINE trial team

**Affiliations:** 1 University Teaching Hospital, Children’s Hospital, Private Bag RW IX, Ridgeway, Lusaka, Zambia; 2 Medical Research Council Clinical Trials Unit at University College London, Institute of Clinical Trials and Methodology, High Holborn, London, WC1V 6LJ UK; 3 Makerere University-John Hopkins University Care Ltd, Kampala, Uganda; 4 India Council of Medical Research, National Institute for Research in Tuberculosis, Chennai, India; 5 Byramjee Jeejeebhoy Government Medical College, Pune, India; 6 Desmond Tutu TB Centre, Stellenbosch University, Cape Town, South Africa; 7 India Institute of Child Health and Hospital for Children, Chennai, India; 8 Radbound University Medical Center, Nijmegen, The Netherlands; 9 University of Cape Town, Cape Town, South Africa; 10 Family Infectious Diseases Clinical Research Unit, Stellensbosch University, Cape Town, South Africa

**Keywords:** Tuberculosis, Efficacy, HIV, Shorter course, Child

## Abstract

**Background:**

Tuberculosis (TB) in children is frequently paucibacillary and non-severe forms of pulmonary TB are common. Evidence for tuberculosis treatment in children is largely extrapolated from adult studies. Trials in adults with smear-negative tuberculosis suggest that treatment can be effectively shortened from 6 to 4 months. New paediatric, fixed-dose combination anti-tuberculosis treatments have recently been introduced in many countries, making the implementation of World Health Organisation (WHO)-revised dosing recommendations feasible. The safety and efficacy of these higher drug doses has not been systematically assessed in large studies in children, and the pharmacokinetics across children representing the range of weights and ages should be confirmed.

**Methods/design:**

SHINE is a multicentre, open-label, parallel-group, non-inferiority, randomised controlled, two-arm trial comparing a 4-month vs the standard 6-month regimen using revised WHO paediatric anti-tuberculosis drug doses. We aim to recruit 1200 African and Indian children aged below 16 years with non-severe TB, with or without HIV infection. The primary efficacy and safety endpoints are TB disease-free survival 72 weeks post randomisation and grade 3 or 4 adverse events. Nested pharmacokinetic studies will evaluate anti-tuberculosis drug concentrations, providing model-based predictions for optimal dosing, and measure antiretroviral exposures in order to describe the drug-drug interactions in a subset of HIV-infected children. Socioeconomic analyses will evaluate the cost-effectiveness of the intervention and social science studies will further explore the acceptability and palatability of these new paediatric drug formulations.

**Discussion:**

Although recent trials of TB treatment-shortening in adults with sputum-positivity have not been successful, the question has never been addressed in children, who have mainly paucibacillary, non-severe smear-negative disease. SHINE should inform whether treatment-shortening of drug-susceptible TB in children, regardless of HIV status, is efficacious and safe. The trial will also fill existing gaps in knowledge on dosing and acceptability of new anti-tuberculosis formulations and commonly used HIV drugs in settings with a high burden of TB. A positive result from this trial could simplify and shorten treatment, improve adherence and be cost-saving for many children with TB.

Recruitment to the SHINE trial begun in July 2016; results are expected in 2020.

**Trial registration:**

International Standard Randomised Controlled Trials Number: ISRCTN63579542, 14 October 2014.

Pan African Clinical Trials Registry Number: PACTR201505001141379, 14 May 2015.

Clinical Trial Registry-India, registration number: CTRI/2017/07/009119, 27 July 2017.

**Electronic supplementary material:**

The online version of this article (10.1186/s13063-018-2608-5) contains supplementary material, which is available to authorized users.

## Background

### Disease setting and context in the study

Of the estimated 10.4 million new tuberculosis (TB) cases globally per annum, approximately one million occur in children in Africa and South East Asia [1]. In Africa, where the TB and HIV epidemics are closely linked, children contribute approximately 30% of incident TB cases [2–4]. Furthermore, in countries with high HIV prevalence, the peak age-prevalence of TB has shifted towards younger adults. These adults are often parents of young children, increasing the exposure of children to TB. Among both HIV-infected children and adults, TB remains a leading cause of death and is the most common opportunistic infection, despite improved access to antiretroviral therapy (ART) [5–7].

Historically, studies of anti-tuberculosis treatment in children have lagged substantially behind adult studies and data regarding TB treatment extrapolated from adults. Children have also been seen to pose little risk of onward transmission. In combination with the challenges in confirming a TB diagnosis in children and the resource constraints, the focus has been on adult trials. The approaches used to treat childhood TB have relied on extrapolation to children of the evidence from clinical trials in adults who have a different spectrum of TB disease and lower bacillary burden [8].

### The spectrum of TB disease in children

The TB disease spectrum observed in children is different compared to adults [9, 10]. Whereas a higher proportion of young children (below 3 years) have disseminated forms of TB, including miliary TB and TB meningitis, [9] primary childhood TB is typically more benign than adult TB. Three-quarters of children have intrathoracic TB, mainly with non-severe forms of intrathoracic lymph node TB [10–12]. Adult forms with pleural effusions and cavitation commonly emerge during adolescence [13].

### TB diagnosis and measuring response to treatment in children

Microbiological confirmation of TB in younger children, who have the highest risk of TB disease, is challenging as they cannot spontaneously produce sputum. In addition, most children with pulmonary (intrathoracic) TB typically have paucibacillary disease. Of the 75% of children with intrathoracic disease, more than 85% have negative-smear microscopy on respiratory samples [11, 12]. Confirmation by culture (the diagnostic ‘gold standard’) is typically made only in approximately 30% of children [11, 14]. Of note, positive culture can also occasionally be obtained in children with normal chest radiographs and a positive tuberculin skin test or history of *Mycobacterium tuberculosis* exposure [15–17]. Therefore, the diagnosis of intrathoracic tuberculosis in children frequently relies on non-specific clinical symptoms and signs, supported by evidence of TB contact and/or TB infection and changes on chest radiography [18].

Data on objective measures of TB treatment response in children are limited. Response to anti-tuberculosis treatment can be evaluated by resolution of clinical symptoms, radiological features and smear and culture conversion in bacteriologically confirmed cases. Radiological resolution has limitations as some features may temporarily worsen and residual changes may persist at the end of treatment [19–22]. Repeat microbiological sampling during therapy may also not be feasible in the majority of children who stop coughing early in treatment. Although well-defined clinical case definitions for research are useful for TB diagnosis and evaluation of treatment response in paediatric studies, [8, 23] there are no published data on the gold standard of TB treatment response, i.e. long-term relapse-free survival.

### Drug doses and fixed-dose formulations of anti-tuberculosis drugs in children

The doses of first-line anti-tuberculosis drugs recommended for children have been based on the same dose per kilogram of body weight that is recommended for adults [8]. In 2010, the WHO revised the doses of the first-line TB drugs in children, increasing daily doses of isoniazid (H) from 5 to 10 mg/kg, rifampicin (R) from 10 to 15 mg/kg, pyrazinamide (Z) from 25 to 35 mg/kg and recommending ethambutol (E) at a dose of 20 mg/kg/day [24]. The change was triggered by a review of the academic literature including pharmacokinetic (PK) studies showing that doses extrapolated from adults do not achieve comparable drug exposures in children [25–29]. With the revised doses it was thought to be possible to attain similar rifampicin, isoniazid and pyrazinamide concentrations to those in adults on standard doses [30–32].

WHO-recommended increased drug doses were difficult to implement in children using the original fixed-dose combination (FDC) RHZ 60/30/150 mg which, in particular, delivered low isoniazid doses [33]. New co-formulated RHZ 75/50/150 mg FDC tablets were launched in 2015 with rapid and wide uptake globally [34]. The new FDC, which we use in the SHINE trial, facilitates achieving the recommended doses [35].

### Anti-TB and antiretroviral treatment interactions in HIV co-infected children

Rifampicin interacts with many antiretroviral drugs necessitating ART adjustment for children. For children ≥ 3 years, efavirenz-based ART is preferred during co-administration of anti-tuberculosis drugs as efavirenz has minimal drug interactions with rifampicin. For children < 3 years on lopinavir/ritonavir-based ART, two options are available: first, super boosting with additional ritonavir to overcome the rifampicin-inducing effect of liver enzymes, and second, changing to three nucleoside reverse transcriptase inhibitors (NRTIs) [36]. However, neither option is optimal. Super boosting with separate liquid ritonavir is frequently not feasible due to its unavailability, complexity of administration, short shelf-life and poor tolerability [37], and triple NRTI ART is less efficacious in viral suppression [38]. Alternative solutions for child-friendly dosing are urgently required for young children [36, 39].

### TB treatment-shortening trials in adults

Early trials in HIV-negative adults with smear-negative TB in Hong Kong and Singapore showed that, in principle, reductions of treatment duration from 6 to 4 months have resulted in relapse rates of only 4% [40–42]. WHO guidelines subsequently recommended 4-month treatment duration for smear-negative TB in 1993, although this decision was soon reversed [43]. Recent trials of treatment-shortening in adults with smear positive TB have demonstrated poorer outcomes associated with shorter regimens [44–46]. However, there is no trial evidence about optimum regimen duration for children.

### Rationale for the trial

The implementation of standard regimens for both adults and children is attractive for TB programmes. The needs of children with TB, who potentially benefit from shorter treatment and who form a considerable proportion of the global disease burden, should be addressed. The costs to children and their families, burden to health services and added toxicity associated with potentially overly long treatment regimens should be also considered. In addition, risks of suboptimal HIV control from drug-drug interactions, the increased pill-burden and its effect on adherence are significant issues for HIV-co-infected children [47] which could be ameliorated by shortening TB treatment.

Treatment outcomes appear considerably better in children than adults with multi-drug resistant (MDR) TB [48], likely because most children have paucibacillary disease. It therefore follows that TB outcomes for shorter first-line regimens could be as good as the longer regimens currently used in adults and children [49, 50]. In addition, there are similarities between minimal TB disease in children and smear-negative, paucibacillary TB in adults, in whom trials of shorter regimens have suggested success. This makes the implementation of trials of shorter treatment of standard first-line regimens conducted in children all the more important, and in different countries where the metabolism of anti-tuberculosis drugs may differ (India/Africa). Finally, revised WHO dosing guidelines for TB drugs aim to achieve substantially higher TB drug exposures in children which should be more efficacious, providing additional rationale to perform this treatment-shortening trial at the time that these dosing guidelines are being implemented globally.

#### Aims

The primary aim of the trial is to determine whether the standard 6-month regimen (8 weeks’ HRZE followed by 16 weeks’ HR) can be reduced with similar efficacy, to 4 months’ (8 weeks’ HRZE followed by 8 weeks’ HR), in African and Indian children with non-severe TB and regardless of HIV status, using revised WHO dosing guidelines and recently introduced new paediatric fixed-dose combination tablets.

The other objectives are:

1. To determine whether the higher WHO-recommended doses of daily first-line anti-TB drugs, given as fixed-dose combination dispersible tablets, and prescribed according to weight bands, give appropriate drug exposures when compared with historical paediatric and adult pharmacokinetic (PK) data
2. To determine, among HIV-infected children, whether currently recommended adjusted strategies and doses of antiretroviral drugs can appropriately overcome the effect of rifampicin at the new higher doses

## Methods

### Trial design

**Fig. 1:**
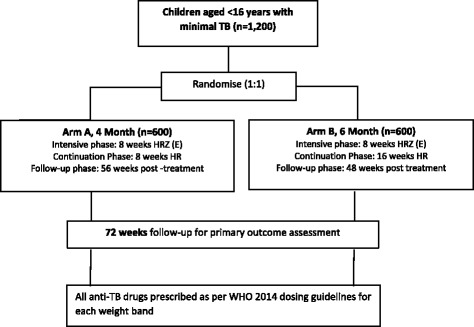
Trial schema

SHINE is an open-label, phase II/III, non-inferiority trial. Participants are randomised in a 1:1 ratio to the experimental 4-month or standard 6-month treatment arm. Both arms have an 8-week intensive phase with rifampicin, isoniazid, pyrazinamide and ethambutol followed by a continuation phase with rifampicin and isoniazid given for 8 weeks in the shorter arm vs 16 weeks in the standard arm (Fig. 1).

### Setting and population

The study recruits children aged < 16 years diagnosed with minimal non-severe TB from diverse populations in three sites in Africa (Cape Town, South Africa; Kampala, Uganda; Lusaka Zambia) and two sites in India (Pune and Chennai). The trial sites were selected based on the expertise of treating children with TB, sufficient clinical trial experience and the potential for recruiting the required number of suitable participants within the agreed recruitment period.

The patient inclusion and exclusion criteria are detailed in Table 1.

**Table 1:** Patient inclusion and exclusion criteria

Inclusion criteria	Exclusion criteria
1. Age 0–16 years 2. Weight ≥ 3 kg 3. Clinician has decided to treat with standard first-line regimen (intensive phase of 4 drugs or 3 drugs as per local practice) 4. Symptomatic but non-severe TB including: a. extrathoracic lymph node TB; intrathoracic uncomplicated (hilar) lymph node TB b. minimal or no parenchymal abnormality on CXR c. smear-negative on gastric aspirate/other respiratory sample Note: GeneXpert may be positive or negative and a negative GeneXpert can be used as a substitute for a negative smear; culture of respiratory sample may be positive or negative; lymph node aspirate may be smear/culture/GeneXpert positive or negative) 5. Not treated for previous TB unless successfully treated > 2 years since last completed treatment 6. Known (or pending confirmation of)HIV status; HIV-infected or HIV-uninfected 7. Willing and likely to adhere to 72 weeks’ follow-up 8. Informed written consent from the parent/legal caregiver(s) and assent in children, as per local Ethics Committee guidance 9. Home address accessible for visiting and intending to remain within the recruitment area for follow-up.	1. Smear-positive respiratory sample 2. Premature (< 37 weeks) *and* aged under 3 months 3. Miliary tuberculosis (TB), spinal TB, TB meningitis, osteoarticular TB, abdominal TB, congenital TB 4. Pre-existing non-tuberculous disease likely to prejudice the response to, or assessment of, treatment, e.g. liver or kidney disease, peripheral neuropathy, cavitation 5. Any known contraindication to taking anti-TB drugs 6. Known contact with drug-resistant adult source case (including mono-resistant TB) 7. Known drug resistance in the child 8. Severely ill 9. Pregnancy

### Definition of minimal (non-severe) TB

Minimal TB in this protocol refers to a form of non-severe, symptomatic, smear-negative TB including extrathoracic lymph node TB, intrathoracic uncomplicated lymph node TB and other non-severe forms of pulmonary TB as per an established classification system [51].

### Endpoints

#### Primary outcome

The main trial outcome for efficacy is categorised as follows:

1. Favourable outcome; TB-free survival at week 72 post randomisation
2. Unfavourable outcomes; a composite of death, reinfection or recurrence
3. Not assessable; will include unknown outcomes at trial completion such as losses to follow-up after completing treatment and clinically well when last seen.

The safety endpoint will be grade 3 and 4 adverse events.

#### Secondary outcomes

1. Mortality
2. Adverse drug reactions within 30 days of completing treatment
3. Relapse/re-infection-free survival including only cases adjudicated to be TB by an independent Endpoint Review Committee
4. HIV viral suppression at weeks 24 and 48 in HIV-infected children
5. Adherence and acceptability
6. Bacterial infections requiring hospitalisation

### Screening and enrolment procedures

The standard screening procedures include: a chest radiograph (CXR) and at least two consecutive respiratory samples including gastric aspirate, expectorated or induced sputum for smear microscopy, Xpert MTB/RIF© (Xpert; Cepheid, Sunnydale, CA, USA), culture and drug-susceptibility testing (DST). In children with extrathoracic cervical lymphadenopathy, lymph node needle aspiration will be undertaken. A Mantoux test or IGRA (interferon-gamma release assay) will be done as available.

At enrolment, a clinical history and physical examination is performed including measurements of weight, height, mid-upper arm circumference, paediatric WHO staging for HIV and signs/symptoms suggestive of TB.

### Randomisation and allocation

Randomisation is undertaken at each site using a web-based system controlled through an authorised username and password. The method of minimisation with a random element is employed. To reduce potential predictability of the randomisation in this open-label study, the minimisation factors are not disclosed to the investigators.

### Trial interventions

Children randomised to the 4-month arm receive rifampicin, isoniazid and pyrazinamide (plus ethambutol where given as part of standard care treatment) for an 8-week intensive phase followed by isoniazid and rifampicin for a continuation phase of a further 8 weeks. In the 6-month arm, children receive the same intensive phase as the other group followed by a continuation phase of 16 weeks.

#### TB treatment products, dosing schedule and treatment supervision

Anti-TB drugs are administered according the WHO dosing recommendations using new FDCs and new weight bands for children weighing < 25 kg (Table 2). Children weighing ≥ 25 kg are treated according to adult guidelines (Table 3).

**Table 2:** Number of tablets to be taken once daily based on WHO guidelines^a^

Weight bands	Intensive phase		Continuation phase
	50H, 75R, 150Z	100E^b^	50H, 75R
3.0–3.9 kg	0.75	0.75	0.75
4.0–7.9 kg	1	1	1
8.0–11.9 kg	2	2	2
12.0–15.9 kg	3	3	3
16.0–24.9 kg	4	4	4
≥ 25.0 kg	Adult formulation and doses	Adult formulation and doses	Adult formulation and doses

*R* rifampicin, *H* isoniazid, *Z* pyrazinamide, *E* ethambutol

^a^H: 10 mg/kg (range 7–15 mg/kg) to a maximum dose 300 mg/day; R: 15 mg/kg (range 10–20 mg/kg) to a maximum dose 600 mg/day; Z: 35 mg/kg (30–40 mg/kg); E: 20 mg/kg (15-25 mg/kg)

^b^100E: additional, separate, single-dose E 100-mg tablets, where this is given as part of local practice, are provided for the intensive phase

**Table 3:** Number of tablets to be taken by children weighing ≥ 25 kg based on WHO adult tuberculosis (TB) guidelines^a^

Weight bands	Intensive phase	Continuation phase
	75H, 150R, 400Z, 275E^b^	75H, 150R
25–36.9 kg	2	2
37–54.9 kg	3	3
55–70.0 kg	4	4

^a^H: 5 mg/kg (range 4–6 mg/kg) to a maximum dose 300 mg/day; R: 10 mg/kg (range 8–12 mg/kg) to a maximum dose 600 mg/day; Z: 25 mg/kg (20–30 mg/kg); E: range 15–20 mg/kg; ^b^Either HRZE or HR fixed-dose combinations with additional Z

Treatment is implemented in accordance with each site’s local practice. Typically this involves daily administration by the child’s carer(s) and documenting ingestion on TB treatment cards provided by the trial.

Adherence to trial medication is assessed by pill counts, specific adherence questions at each 4-weekly visit, as well as the number of doses recorded on the TB treatment card.

#### Protocol treatment discontinuation

Protocol treatment discontinuation is considered under any of the following circumstances:

1. Unacceptable toxicity or adverse event

2. Disease progression necessitating change of regimen (e.g. with drug-resistant TB or complication of disease)

3. Withdrawal of consent for treatment or participation by the participant’s carer/guardian

4. Participant’s DST-identifying drug resistance, including isoniazid mono-resistance

The children whose protocol treatment is discontinued remain in the trial for the purpose of follow-up and data analysis (unless the carer withdraws consent). Data are kept and included for participants who stop follow-up early, up to the point of exit.

#### Non-trial treatment

HIV-infected children enrolled in the trial are provided with ART in accordance with local country programmes including alternative ART drugs regimens indicated for concomitant use with rifampicin during the duration of the TB treatment. All HIV-infected children with TB receive co-trimoxazole prophylaxis as per local guidelines.

### Trial assessments and follow-up

**Fig. 2:**
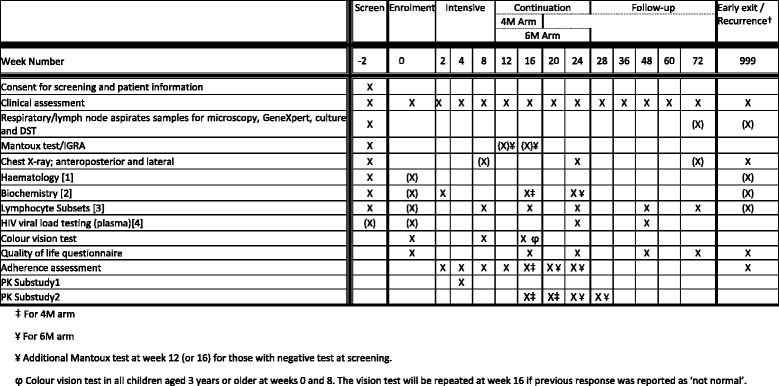
SHINE trial assessment and follow-up schedules

All participants are seen at screening, enrolment/randomisation and then at weeks 2, 4, 8, 12, 16, 20, 24, 28, 36, 48, 60 and finally at week 72, when they will exit the trial (Fig. 2).

A symptom checklist is performed at each visit to detect inter-current illnesses, new TB exposure including DR-TB, TB treatment failure/recurrence or relapse or adverse events to TB drugs (and ART if applicable). Respiratory samples for TB assessment and blood samples for biochemistry and haematology, as well as lymphocyte subsets and HIV1 viral load in HIV-infected children, are obtained as summarised in Fig. 2. Relevant samples are stored for pharmacogenomics, biomarkers and hair PK substudies at pre-specified time points (Fig. 2). A quality of life questionnaire, adapted for younger children from a standardised EQ-5D questionnaire [52], is administered at weeks 0, 16, 24, 48 and 72.

Participants are expected to attend all scheduled visits unless agreed in advance with the study team. Additional study visits are arranged when a participant misses a clinic visit or whenever clinical care is required.

#### Procedures for assessment of TB diagnosis and treatment response

TB diagnosis and treatment response will be adjudicated by an Endpoint Review Committee (ERC) with independent chair and membership. The ERC will review the endpoints (treatment failure or recurrence) and cause of death against standard criteria [23], blinded to treatment allocation, based on all available clinical, microbiological and molecular data obtained from the follow-up assessments and procedures. TB disease severity at diagnosis will be ascertained using Wiseman criteria [51].

CXRs are repeated at week 24, for suspected treatment failure or TB recurrence, and if clinically indicated. All CXRs will be prospectively read by the study site clinician to check eligibility for enrolment and retrospectively reviewed centrally by independent experts blind to treatment allocation using a standardised approach. Where available, Mantoux/IGRA is repeated at week 12 or 16 if the initial test was negative or missed at screening.

At each visit a clinical examination for TB signs and symptoms and new TB exposure is conducted to identify children with possible treatment failure or recurrence. Repeat specimens for smear microscopy and culture for *M. tuberculosis* are collected if clinically indicated. For children with culture-positive respiratory specimens, repeat TB microscopy and culture are attempted every 2 months until culture conversion. Lymph node needle aspiration is not routinely repeated during follow-up.

#### Procedures for assessing safety

Symptoms checklists and laboratory investigations are performed as part of safety monitoring at predetermined intervals (Fig. 2). In the case of adverse events, additional tests are done as clinically indicated.

To monitor for the potential ocular toxicity with ethambutol use, colour-vision testing is done at screening and at week 8 for children aged 3 years and older, with repeat testing at 16 weeks only in those patients with abnormal results. Children with obvious symptoms at any time within the intensive phase of treatment are referred to an ophthalmologist for further testing as appropriate. In the event of confirmed ocular toxicity, ethambutol is stopped.

For girls of reproductive age, or who have started menses, a pregnancy test is performed at screening. Any girl found to be pregnant is not eligible for the trial. Girls who become pregnant during the trial will be closely monitored and followed for treatment and pregnancy outcome.

## Substudies

### Pharmacokinetic (PK) studies

The trial includes two PK substudies. The first includes 65 children and describes the PK parameters of rifampicin, isoniazid and pyrazinamide when administered in the new FDC according to WHO weight-band doses in children weighing < 25 kg, or the adult formulation for children 25 to < 37 kg [18]. Ethambutol will be measured in the subset of children treated with this fourth drug.

The second substudy evaluates PK interactions between anti-TB and antiretroviral drugs (efavirenz, lopinavir, ritonavir, nevirapine, abacavir and zidovudine) in up to 60 HIV-infected children on ART. Children have cross-over intensive PK undertaken during the continuation phase and 4 weeks after stopping anti-TB treatment.

For both studies, seven blood samples (0.8 ml each) are collected before drug intake and then at 1, 2, 4, 6, 8 and 12 h after drug intake for intensive PK.

### Hair PK sub-study

Novel assays to measure concentrations of two TB drugs, isoniazid and pyrazinamide, in small hair samples have been developed and validated. These assays have the potential to monitor adequate drug adherence and exposure and need further investigation. In the SHINE study, small hair samples are being collected in around 600 consenting participants to evaluate hair concentrations of isoniazid and pyrazinamide as surrogates of adherence and predictors of TB treatment outcomes (e.g. treatment success, recurrence and death) among HIV-infected and uninfected children.

### Health economics

Economic evaluation of the alternative interventions will be undertaken. Resource use data are collected in the trial on health care visits, hospitalisations, TB and HIV medications, concomitant medications, and diagnostic tests. Estimates of the total costs of the two treatment strategies from a health sector perspective will be made using nationally representative unit costs combined with resource use data. Health outcomes will be estimated in terms of life years gained (LYGs), quality-adjusted life years (QALYs) and disability-adjusted life years (DALYs). The adapted quality of life questionnaire [52] will be combined with existing tariffs to estimate QALYs. Existing DALY weights for TB and TB-free survival will be used.

The nature of the economic analyses will largely depend upon the trial results. If the shortened regimen arm is non-inferior, and is not associated with any additional costs, this will indicate its cost-effectiveness. The cost savings from adopting this strategy at the national level will then be estimated based upon national estimates of childhood TB incidence. If the longer regimen arm is shown to be more effective, a cost-effectiveness analysis will be undertaken to investigate whether this represents ‘value for money’ to each of the health care systems in terms of the cost per QALY/DALY/LYG, and estimates of the opportunity costs of resources required to fund the intervention.

### Social science

The substudy evaluates palatability and acceptability of the new FDC using acceptability questionnaires and semi-structured interviews in health facilities and at the homes of a subset of the South African trial population. Challenges with administration of the anti-TB drugs and differences between clinical advice and practice, as well as changes in administration and acceptability over the period of treatment, will be described.

### Statistical considerations

#### Sample size and power

It is expected that TB recurrence (relapse or re-infection) and mortality rates will differ by HIV status and co-administration of ART. Based on estimates from the ARROW trial (Uganda and Zimbabwe) [53] and routine data from paediatric HIV clinics in South Africa, we estimate mortality in children HIV-negative, HIV-infected children on and not on ART to be 2%, 5% and 7%, respectively, over 18 months. Corresponding % estimates for TB recurrence are 3%, 4% and 6%, respectively, in these three groups. We assume that the proportion of HIV-infected children will be approximately 35% maximum (averaged across sites), of whom the majority will be on ART at the start of TB therapy. Using these estimates, the overall rate of TB recurrence or death was estimated at approximately 8%.

#### Non-inferiority margin

In discussion with study investigators, 6% was considered a reasonable margin of non-inferiority for the experimental arm for the endpoint of relapse or re-infection-free survival. This has been used in previous treatment-shortening phase III trials in adult smear-positive TB [45, 46].

Based on these assumptions, the total number of evaluable children required to demonstrate non-inferiority between the short-regimen arm and the control arm (90% power, one-sided 97.5% confidence intervals (CIs)), would be 860 across two arms. Based on experience from other phase III TB trials, 10% loss-to-follow-up was considered reasonable, resulting in a total of 956 children.

It is expected that a proportion of enrolled children treated for TB will not actually have TB, thus reducing the recurrence rate and increasing the chance of demonstrating non-inferiority. For this reason, although the primary analysis will include all randomised children (with assessable outcomes), a key secondary analysis will exclude children (assumed 20%) considered by the independent Blinded-endpoint Review Committee not to have TB at enrolment.

Due to the importance of this analysis the trial will be powered to demonstrate non-inferiority also in this sub-population, resulting in a total of 1200 children to be enrolled.

#### Analysis plan

There will be two main analysis populations: the modified intention-to-treat (MITT) and per-protocol (PP) analysis populations. While both of these will be considered primary in order to declare non-inferiority, consistent results should be obtained from key subgroup analyses (confirmed TB cases, both MITT and PP) and sensitivity analyses, although not all of these will necessarily need to satisfy the strict definition of non-inferiority.

The MITT analysis will include all randomised participants excluding those withdrawn from treatment because of a protocol violation at enrolment (late exclusions from the study, based on data collected prior to randomisation). The PP analysis will be restricted to participants who have received an adequate amount of study treatment. Any further exclusions in both MITT and PP populations will be detailed in the statistical analysis plan.

#### Definition of adequate treatment

The definition of adequate treatment sets limits both for the amount of treatment missed in each treatment phase and the amount of treatment missed overall. This definition of adequate treatment has been used in adult TB trials and is based on receiving at least 80% of the prescribed doses within a pre-specified time frame [45].

#### Primary efficacy analysis

The primary efficacy analysis will be the difference in proportions of the unfavourable outcomes (proportion with unfavourable outcome on the 4-month regimen arm less the proportion with unfavourable outcome on the 6-month regimen) adjusted for the minimisation factors.

Non-inferiority will be assessed using the upper bound of the one-sided 97.5% CI for this difference. If the upper bound of this CI is less than 6% (the margin of non-inferiority) the 4-month regimen will be considered non-inferior to the 6-month regimen.

#### PK substudies analysis plan

Non-linear mixed effects (NLME) models will be used to describe the population PK of the TB and ART drugs taking account of age, weight, race (African/Asian) and actual dose. In addition to describing the PK for each drug, these models would account for any non-linearity in the PK related to dose.

Differences in the Area Under the Curve (AUC) by age, HIV status, and between Asian vs African would be determined using model-based individual post-hoc Bayesian estimates. The geometric mean ratios of the post-hoc estimates of the AUCs for the TB drugs in the FDC compared to single drugs in regulatory approved products will be computed and evaluated against bioequivalence criteria.

### Safety reporting

#### Adverse events and adverse reactions

The definitions of adverse events (AE) and adverse reactions (AR) outlined in the EU Directive 2001/20/EC Article 2 based on the principles of ICH GCP apply to this trial protocol (Table 4) [54].

**Table 4:** Safety reporting definitions

Term	Definition
Adverse event (AE)^a^	Any untoward medical occurrence in a patient or clinical trial subject to whom a medicinal product has been administered including occurrences that are not necessarily caused by or related to that product
Adverse reaction (AR)	Any untoward and unintended response to an investigational medicinal product related to any dose administered
Unexpected adverse reaction (UAR)	An adverse reaction, the nature or severity of which is not consistent with the information about the medicinal product in question set out in the Summary of Product Characteristics (SPC) or Investigator Brochure (IB) for that product
Serious adverse event (SAE) or serious adverse reaction (SAR) or suspected unexpected serious adverse reaction (SUSAR)	Respectively, any adverse event, adverse reaction or unexpected adverse reaction that: Results in death Is life-threatening Requires hospitalisation or prolongation of existing hospitalisation Results in persistent or significant disability or incapacity Consists of a congenital anomaly or birth defect Is another important medical condition

^a^AEs will include; an exacerbation of a pre-existing illness, an increase in frequency or intensity of a pre-existing episodic event or condition, a condition (even though it may have been present prior to the start of the trial) detected after trial drug administration or continuous persistent disease (or a symptom present at baseline) that worsens following administration of the study treatment

Conditions exempted from AEs will include; medical or surgical procedures (the condition that leads to the procedure is the adverse event), pre-existing disease or a condition present before treatment that does not worsen, hospitalisations where no untoward or unintended response has occurred (e.g. elective cosmetic surgery, social admissions) or overdose of medication without signs or symptoms

The investigational medicinal products in this trial will be rifampicin, isoniazid, pyrazinamide and ethambutol. ARs include any untoward or unintended response to any of these drugs.

All AEs meeting the standard definitions above are reported, regardless of their relationship to TB. The seriousness of the events is assessed using the ICH-GCP definitions, and severity is determined in accordance with the 2014 Division of AIDS toxicity grading scale [55]. The causality and expectedness of all serious events or reactions in relation to the trial drugs is categorised as unrelated, unlikely, possibly, probably and definitely related, and for the latter three categories as expected and unexpected based on the Summary of Product Characteristics.

All SAEs, grade 3/4 adverse reactions and notable events (ocular toxicities, pregnancies and bacterial infections requiring hospitalisation) are notified to the Medical Research Council Clinical Trials Unit (MRC CTU) within one working day of the investigator becoming aware of the event. Other reportable events are reported within seven working days. The investigators at each site are also responsible for the onward reporting of adverse events to their ethics and regulatory bodies, as per their local reporting requirements. MRC CTU is responsible for annual onward reporting of a line listing of events to the trial sponsor, University College London (UCL). Investigators are responsible for reporting all SAEs, notable events and other adverse events meeting their reporting requirements that occur from the time of randomisation until 30 days after the last protocol treatment administration.

### Quality assurance, data management and monitoring

UCL is responsible for trial oversight and ensuring that arrangements are in place for adequate management, monitoring, analysis and reporting of the trial.

Quality assurance and quality control are based on a formal risk assessment which leads to the development of the trial quality management plan implemented by the sponsor. On-site monitoring and data validation and assessment of adherence to International Conference on Harmonisation-Good Clinical Practice (ICH-GCP) principles is regularly conducted by independent local monitors at each site and periodically directly by MRC CTU on behalf of the sponsor. Each site is responsible for its own data entry and local trial management.

### Trial governance

The trial is overseen by a number of committees each with distinct functions and membership. The Trial Steering Committee (TSC) consists of the chief investigator, site principal investigators and independent members. The TSC provides overall supervision for the trial and advice through its independent chair. The ultimate decision for the continuation of the trial lies with the TSC.

The Independent Data Monitoring Committee (IDMC) reviews confidential, accumulating data for the trial produced by the trial statisticians. The functions and frequency of its meeting are dictated by the DMC charter. The IDMC can recommend premature closure or reporting of the trial.

The Endpoint Review Committee (ERC), blinded to the trial arm, will determine the primary endpoint classification for all participants as favourable, unfavourable or unassessable. In addition, the ERC will examine evidence and adjudicate causes of death based on all available sources of data and determine the TB diagnosis at enrolment across sites for the key secondary analysis of those with definitive TB [23].

## Discussion

SHINE is the first large-scale paediatric trial that will test whether 4-month therapy is as effective as the standard 6-month course in children with non-severe TB. Recently, three large multicentre trials to determine whether fluoroquinolone-containing regimens can shorten treatment from 6 to 4 months did not demonstrate non-inferiority of the 4-month regimens in adults with drug susceptible TB [44–46]. In SHINE, unlike in these adult trials, smear-negative children with non-severe paucibacillary disease will use revised doses of first-line anti-TB drugs. In addition, the trial will also demonstrate whether the PK parameters and safety profile of the new dosing recommendations for rifampicin, isoniazid, pyrazinamide and ethambutol across populations of Indian and African children are adequate. As TB often occurs alongside HIV infection, SHINE will provide answers for optimal anti-TB drug dosing in HIV co-infected children on concomitant ART. The nested economic and social science substudies enable SHINE to rapidly translate findings into budget relevant, patient-oriented, and context-sensitive policy guidance.

TB studies in children have long been neglected owing to the perception that paediatric TB is not a public health priority. Paediatric TB trials, in particular, face challenges because of difficulties in confirming TB bacteriological conversion as a study endpoint [8]. While acknowledging the limitations associated with the establishment of paediatric TB diagnosis and evaluating endpoints, both clinically diagnosed and bacteriologically confirmed cases of non-severe TB will be included, reflecting the pragmatic approaches utilised in routine clinical settings where children are diagnosed, treated and monitored predominantly using clinical and radiological parameters. The TB diagnoses and primary endpoints will be adjudicated by an independent ERC, blinded to the study arms, against the standard paediatric TB research consensus criteria [23, 51, 56]. The trial will utilise a centralised imaging review by independent paediatric TB radiology experts to assist with decisions on the endpoints. Although the main primary analysis will include all randomised children, it is anticipated that some children might not have TB, thus contributing to a non-inferiority outcome. To overcome this, the study was powered for demonstration of non-inferiority in a key subpopulation considered to have TB by the ERC.

The trial includes HIV-infected children as current evidence suggests no association between treatment duration and outcomes in patients on ART receiving rifampicin-containing regimens [57]. According to WHO guidance and existing programmatic approaches, HIV-infected and uninfected children should be treated for the same duration [18, 36]. Ethically, the risk profile of the trial will be similar to existing recommended approaches in routine clinical practice for TB treatment in children, regardless of HIV status.

Economic analyses will assess the cost-effectiveness of reducing treatment from 6 months to 4 months while the social science analyses will evaluate the palatability and acceptability of using the recently implemented paediatric anti-tuberculosis FDCs.

SHINE will confirm whether or not treatment-shortening of drug susceptible TB in children is efficacious and safe. It will also fill existing gaps in knowledge on dosing of the new anti-TB formulations and commonly used HIV drugs in settings with a high burden of childhood TB. A positive result from this trial (i.e. if 4 months is shown to be non-inferior to 6 months) will be to simplify treatment, to potentially improve adherence and to reduce treatment costs for a large proportion of children with TB (Additional file 1).

### Trial status

The first child was enrolled to SHINE in July 2016 and recruitment is due to finish by the end of June 2018.

## Additional file

Additional file 1: SPIRIT 2013 Checklist: recommended items to address in a clinical trial protocol and related documents. (DOC 125 kb)

## Abbreviations

3TC: Lamivudine
ABC: Abacavir
AE: Adverse event
ALT: Alanine aminotransferase
AR: Adverse reaction
ART: Antiretroviral therapy
ARV: Antiretroviral
AUC: Area under the curve
CTU: Clinical trials unit
CXR: Chest x-ray
DALY: Disability-adjusted life years
DST: Drug-susceptibility test
E: Ethambutol
EFV: Efavirenz
ERC: Endpoint Review Committee
FDC: Fixed-dose combination
GCP: Good clinical practice
H: Isoniazid
Hb: Haemoglobin
HIV: Human immunodeficiency virus
ICH: International Conference on Harmonisation
IDMC: Independent Data Monitoring Committee
IGRA: Interferon-gamma release assay
IRB: Institutional review board
ISRCTN: International standard randomised controlled trial number
LPV: Lopinavir
LPV/r: Lopinavir with ritonavir
LYG: Life years gained
MDR: Multiple drug resistant tuberculosis
MITT: Modified intention-to-treat
MRC: Medical Research Council
NNRTI: Non-nucleoside reverse transcriptase inhibitor
NRTI: Nucleoside reverse transcriptase inhibitor
NVP: Nevirapine
PK: Pharmacokinetics
PP: Per protocol
QALY: Quality-adjusted life year
R: Rifampicin
SAE: Serious adverse event
TB: Tuberculosis
TSC: Trial Steering Committee
UCL: University College London
WHO: World Health Organisation
ZDV: Zidovudine
